# Increased Oxidative Stress in Acute Myeloid Leukemia Patients after Red Blood Cell Transfusion, but Not Platelet Transfusion, Results Mainly from the Oxidative/Nitrative Protein Damage: An Exploratory Study

**DOI:** 10.3390/jcm10071349

**Published:** 2021-03-25

**Authors:** Kamila Czubak-Prowizor, Jacek Trelinski, Paulina Stelmach, Piotr Stelmach, Agnieszka Madon, Halina Malgorzata Zbikowska

**Affiliations:** 1 Department of General Biochemistry, Faculty of Biology and Environmental Protection, University of Lodz, Pomorska 141/143, 90-236 Lodz, Poland; halina.zbikowska@biol.uni.lodz.pl; 2 Department of Cytobiology and Proteomics, Medical University of Lodz, Mazowiecka 6/8, 92-215 Lodz, Poland; 3 Department of Coagulation Disorders, Medical University of Lodz, Ciolkowskiego 2, 93-510 Lodz, Poland; jacek.trelinski@umed.lodz.pl; 4 Department of Haematology, Medical University of Lodz, Copernicus Memorial Hospital, Ciolkowskiego 2, 93-510 Lodz, Poland; p.stelmach83@gmail.com (P.S.); p.stelmach84@gmail.com (P.S.); 5 Laboratory of Transfusion Serology and Blood Bank, Copernicus Memorial Hospital, Pabianicka 62, 93-513 Lodz, Poland; agnieszka-madon@wp.pl

**Keywords:** acute myeloid leukemia, blood platelet, red blood cell, transfusion, oxidative stress markers

## Abstract

Chronic oxidative stress (OS) can be an important factor of acute myeloid leukemia (AML) progression; however, there are no data on the extent/consequence of OS after transfusion of packed red blood cells (pRBCs) and platelet concentrates (PCs), which are commonly used in the treatment of leukemia-associated anemia and thrombocytopenia. We aimed to investigate the effects of pRBC/PC transfusion on the OS markers, i.e., thiol and carbonyl (CO) groups, 3-nitrotyrosine (3-NT), thiobarbituric acid reactive substances (TBARS), advanced glycation end products (AGE), total antioxidant capacity (TAC), SOD, GST, and LDH, in the blood plasma of AML patients, before and 24 h post-transfusion. In this exploratory study, 52 patients were examined, of which 27 were transfused with pRBCs and 25 with PCs. Age-matched healthy subjects were also enrolled as controls. Our results showed the oxidation of thiols, increased 3-NT, AGE levels, and decreased TAC in AML groups versus controls. After pRBC transfusion, CO groups, AGE, and 3-NT significantly increased (by approximately 30, 23, and 35%; *p* < 0.05, *p* < 0.05, and *p* < 0.01, respectively) while thiols reduced (by 18%; *p* < 0.05). The PC transfusion resulted in the raise of TBARS and AGE (by 45%; *p* < 0.01 and 31%; *p* < 0.001), respectively). Other variables showed no significant post-transfusion changes. In conclusion, transfusion of both pRBCs and PCs was associated with an increased OS; however, transfusing the former may have more severe consequences, since it is associated with the irreversible oxidative/nitrative modifications of plasma proteins.

## 1. Introduction

Acute myeloid leukemia (AML) is a hematologic malignancy distinguished by a rapid, uncontrolled clonal growth of myeloid lineage cells in the bone marrow. In AML patients, severe anemia and a bleeding risk remain significant clinical problems, resulting from the infiltration by leukemic cells in the bone marrow and the suppressive effect of a cytotoxic drug therapy [1]. In the treatment of deep, leukemia-associated anemia (hemoglobin (Hgb) level below 7 g/dL) or profound thrombocytopenia (platelet (PLT) count below 20,000/µL) transfusion of packed red blood cells (pRBCs) and platelet concentrates (PCs) are regularly used [2].

Blood transfusion is often associated with some risk factors, due to a genetic difference between the transfused blood cells and the tissues of the recipient’s system, as well as the possibility of transferring a plethora of biologically active molecules along with the blood components [3,4]. During their blood bank storage, in the presence of the additive solutions (up to 42 days, at 4 ± 2 °C), red blood cells (RBCs) undergo progressive aging-associated changes and oxidative damage, which are collectively referred to as the storage lesion [3]. Oxidative stress (OS) is an undesirable phenomenon that is well documented to occur in the stored pRBCs, in the majority due to the Hgb release and breakdown [3,5]. Platelets are stored for up to 5–7 days, at 22 °C, the PLT storage lesion is mainly coordinated by platelet-activating signals, which ultimately lead to platelet aggregation and release of granular contents and expression of sequestered membrane proteins (selectin-P, CD40L) on the outer surface [4]. Procedures such as leukocyte filtration and irradiation before storage also lead to a different degree of the lesion to RBCs/PLTs [6].

Evidence for chronic OS, caused by the increased production of reactive oxygen/reactive nitrogen species (ROS/RNS) and/or depletion of the antioxidant defense systems, has been found in several hematopoietic malignancies including AML [7,8]. Some reports indicate that relapse in this disease is associated with increased OS markers within the leukemic blasts, suggesting that ROS production may be an important factor of AML progression [8]. Transfusion dependency at diagnosis and transfusion intensity during initial chemotherapy were associated with poorer outcomes in AML adult patients [1], but the underlying molecular mechanisms are not well understood. The current data on the post-transfusion OS in pRBC recipients are controversial and limited to studies on few selected patient groups (neonatal, critically ill, and receiving chronic transfusion therapy) [9,10,11,12], while the impact of PC transfusion on the redox imbalance has not been investigated yet.

This exploratory study aim was to investigate the effects of pRBC or PC transfusions on the OS level in plasma of AML patients. A wide panel of the OS markers, such as the concentrations of total thiols, carbonyl (CO) groups, 3-nitrotyrosine (3-NT), thiobarbituric acid reactive substances (TBARS) and advanced glycation end products (AGE), total antioxidant capacity (TAC), activities of superoxide dismutase (SOD) and glutathione transferase (GST), and lactic dehydrogenase (LDH), before and 24 h post-transfusion of the blood component, were assessed. The hypothesis for this study was that OS increases following blood transfusion. To our knowledge, this is the first study to examine the relationship between blood transfusion and OS in AML patients.

## 2. Materials and Methods

### 2.1. Chemicals

2,2′-azinobis-(3-ethylbenzothiazoline-6-sulfonic acid) (ABTS), 5,5′-dithio-bis-(2-nitrobenzoic acid) (DTNB), 6-hydroxy-2,5,7,8-tetramethylchromane-2-carboxylic acid (Trolox), hydrogen peroxide, nicotinamide adenine dinucleotide (NADH), reduced glutathione (GSH), 1-chloro-2,4-dinitrobenzene (CDNB), sodium pyruvate, thiobarbituric acid (TBA), trichloroacetic acid (TCA), 2,4-dinitrophenylhydrazine (DNPH), anti-DNP antibody produced in rabbit, anti-rabbit IgG (whole molecule)–peroxidase antibody produced in goat, SOD Determination Kit, and Sigma Fast™ OPD Tablet Sets were purchased from Sigma-Aldrich Chemical Co., Warsaw, Poland. Pierce™ BCA Protein Assay Kit and rabbit anti-goat IgG secondary antibody biotin were from Thermo Fisher Scientific, Waltham, MA, USA. OxiSelect™ Advanced Glycation End Product (AGE) Competitive ELISA Kit was obtained from Cell Biolabs, San Diego, US. Anti-nitro tyrosine antibody and streptavidin (HRP) were purchased from Abcam, Cambridge, Great Britain. Other chemicals, all of the analytical grade, were obtained from POCh, Gliwice, Poland.

### 2.2. Study Design and Patient Selection

The study group was recruited between March 2017 and December 2018, among adult patients with AML (general condition 0–3 in the ECOG (Eastern Cooperative Oncology Group) scale) during a hospitalization at the Department of Hematology of the Copernicus Memorial Hospital in Lodz (Poland). In this exploratory study, 52 patients, diagnosed with AML on the basis of the diagnostic criteria of the WHO classification from 2008, participated in the study. Of these, 27 patients were transfused with the irradiated leukocyte-reduced pRBCs, and 25 patients were treated with the irradiated leukocyte-reduced PCs. Each patient could be included only one time.

Criteria for exclusion from the study were as follows: renal failure (GFR (glomerular filtration rate) <30 mL/min), liver failure (the activity of alanine aminotransferase (ALT) and/or aspartate aminotransferase (AST) three-fold upper limit of normal), autoimmune diseases, endocrine disorders, and other malignancies. During hospitalization, patients were not administered substances with antioxidant properties.

The control group comprised 43 healthy volunteers. The group of AML patients was comparable (homogeneous) with the control group in terms of age and sex. The mean age of the control group was 54.5 ± 9.5 years (ranged from 22 to 71 years), while the median was 55 years; women constituted about 45% of this group, men the remaining 55%. The volunteers were not diagnosed with any chronic disease or other diseases that could affect the analyzed parameters of oxidative stress.

### 2.3. Blood Collection and Isolation of Plasma

Venous blood from AML patients was drawn twice, before (approximately 30 min) and after (24 h) transfusion of the blood component. Blood samples were collected into the S-Monovette^®^ blood collection tubes containing 3.8% citrate (Sarstedt, Nümbrecht, Germany) (blood to citrate ratio was 9:1). The collected blood was immediately (within 15 min after blood collection) centrifuged (10 min, 1800× *g*, 20 °C) to obtain citrated plasma. Each plasma sample was aliquoted (to avoid freeze-thaw cycles), frozen at −32 °C, and stored at this temperature until assayed, but no longer than a week after blood collection.

### 2.4. Measurement of the Oxidative Stress-Related Markers

All analyzes were performed in 96-well microplates and absorbances were measured in the plate reader (SPECTROstar^®^ Nano BMG LABTECH GmbH; Offenburg, Germany).

#### 2.4.1. Protein Oxidative/Nitrative Modifications

The total plasma thiols were determined with Ellman’s reagent (DTNB) [13,14]. Briefly, 10 mM phosphate buffer (pH 8.0) containing 10% SDS (20 µL) was mixed with plasma (20 µL). Then, 160 µL of the same buffer was added and the absorbance (A_0_) was measured at 412 nm. Next, 0.04% DTNB in 10 mM phosphate buffer (pH 8.0) was added (16.6 µL). After incubation (1 h, 37 °C), the absorbance at 412 nm (A_1_) was recorded. The concentration of thiols was calculated based on the absorbance difference (A_1_–A_0_) using the molar extinction coefficient (ε = 13,600 M^−1^ cm^−1^).

The protein CO groups were detected by the enzyme-linked immunosorbent assay (ELISA) according to Buss et al. [15] with modifications by Alamdari et al. [16]. Wells of a 96-well microplate were coated with plasma (10 µg protein/mL; 100 µL) and incubated overnight at 4 °C. Pierce™ BCA Protein Assay Kit (Thermo Fisher Scientific; Waltham, MA, USA) was used to measure the total plasma protein concentration. Plasma samples adsorbed to wells were reacted with 0.0024% DNPH solution (200 μL, pH 6.2) and probed with the rabbit anti-DNP primary antibody (200 µL; 60 min) followed by a second anti-rabbit antibody conjugated with horseradish peroxidase (200 µL, 60 min). More details on the method were previously described [17].

Detection of 3-NT in the plasma proteins was carried out by a competitive ELISA, according to Khan et al. [18], as described previously [19] with some modifications. Wells of a 96-well microplate were coated with nitrated human fibrinogen (3-NT-Fg; standard antigen, 100 μL/well) at a concentration of 1 µg/mL and incubated overnight at 4 °C. Wells were washed, blocked with 1% milk, and washed again. Blood plasma (free antigen) was pre-incubated (30 min, ambient temperature) with the diluted (1:40,000) anti-3-NT antibody (plasma:antibody was 1:1), and was then added to the wells (incubation: overnight at 4 °C). Next, wells (after washing) were probed with the biotinylated secondary antibody (100 μL; 60 min; 1: 2000) followed by the streptavidin complex with biotinylated horseradish peroxidase (100 μL; 60 min, 1:10,000). OPD substrate solution (Sigma Fast™ OPD Tablet Sets) was applied, and after incubation (10 min), the reaction was stopped by the addition of 40% sulfuric acid (50 μL). The absorbance was measured at 490 nm. The calibration curve (0.0018–2.0 µg/mL) was prepared by serial dilutions of nitrated Fg. The nitration was performed by treating the solution of human Fg (2 mg/mL) with 1 mM peroxynitrite. The 3-NT concentration in Fg was determined spectrophotometrically by measuring the absorbance at 430 nm, after which it was calculated using the molar extinction coefficient (ε = 4400 M^−1^ cm^−1^).

#### 2.4.2. Lipid Peroxidation

Lipid peroxidation was quantified by measuring the concentration of TBARS [14,20]. Briefly, equal volumes of plasma and 15% trichloroacetic acid (TCA) containing 0.25 M HCl were mixed and incubated in an ice bath (30 min). After centrifugation (7000× *g*, 10 min), the supernatant (200 µL) was collected into clean tubes and 200 µL 0.37% TBA containing 0.25 M HCl was added. Samples were incubated (10 min, 100 °C), and after cooling, equal volumes of clear solutions were transferred to wells on a 96-well microplate. The absorbance was measured at 535 nm, the TBARS concentration was calculated using the molar extinction coefficient for MDA (ε = 156,000 M^−1^ cm^−1^).

#### 2.4.3. Advanced Glycation End Products

The content of AGE was evaluated using OxiSelect™ Advanced Glycation End Product (AGE) Competitive ELISA Kit (Cell Biolabs; San Diego, CA, USA). The assay was performed according to the manufacturer protocol. An aliquot of 50 µL of plasma (or AGE-BSA standard) was applied. A standard curve for AGE-BSA was prepared in the concentration range (0–100 μg/mL).

#### 2.4.4. Total Antioxidant Capacity

TAC was assessed by the method of Erel [21]. The details of the method were previously described [22]. A calibration curve in the concentration range of 0.008–1.0 μmol/mL was prepared using Trolox, a reference antioxidant. The TAC of plasma was calculated on the basis of differences in absorbance A_1_–A_0_ and the standard curve.

#### 2.4.5. SOD Activity

SOD activity was determined using SOD Determination Kit (Sigma-Aldrich; St. Louis, MO, USA). The assay was performed according to the manufacturer protocol. The SOD activity was expressed as the inhibition rate (%).

#### 2.4.6. GST Activity

GST activity was estimated as described by Habig et al. [23], using 1-chloro-2,4-dinitrobenzene (CDNB) and GSH as substrates. The GST activity was calculated using the millimolar extinction coefficient for 2,4-dinitrophenyl-S-glutathione (ε = 9.6 mM^−1^ cm^−1^).

#### 2.4.7. LDH Activity

LDH activity was estimated by the method of Wroblewski and La Due [24] and it was calculated in international units per liter (IU/L).

#### 2.4.8. Statistical Analysis

All results are presented as mean values ± standard error (SE). Each plasma sample was performed in triplicate. The normal distribution of the results was analyzed using a Shapiro–Wilk test. Then, homogeneity of variance was tested using the Brown-Forsythe test. In the next step, a two-way analysis of variance (ANOVA) followed by Tukey’s post-hoc test was performed to assess the significance of differences between values. All data were analyzed using statistical software StatSoft Inc. “Statistica” v. 13.1. *p* < 0.05 was accepted as statistically significant. All presented figures were prepared using GraphPad Prism 5 Software.

## 3. Results

Fifty-two patients were included, of which 27 were transfused with pRBCs (24 patients received two pRBC units, 3 patients received one pRBC unit) and 25 were transfused with PCs (all received one unit). Detailed characteristics of the patients enrolled in the study are presented in Table 1. The storage time of the pRBCs was in majority between 2 to 10 days; only four of the transfused units had been stored longer (up to 11–26 days). Therefore, it was not possible to assess the impact of the RBC storage duration on the OS level. Likewise, only five patients received “older” PCs (stored more than 3 days).

**Figure 1:**
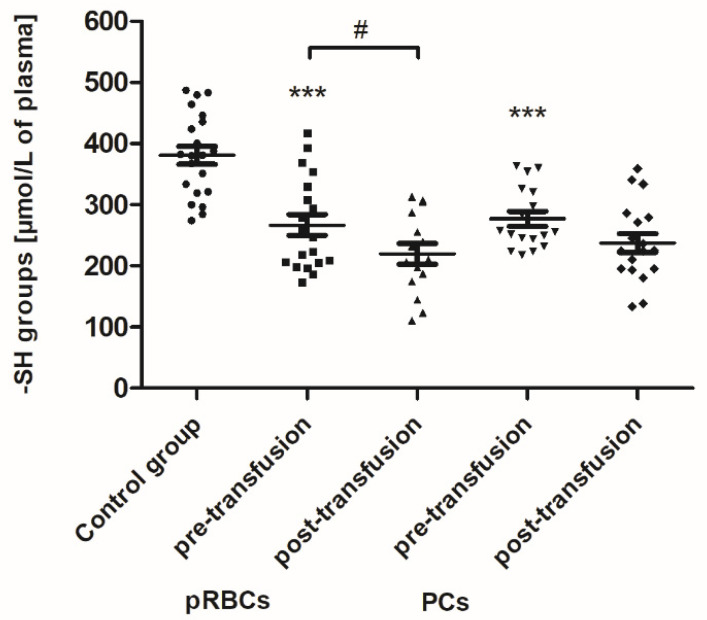
The concentration of total thiols in the plasma of healthy volunteers (control group) and AML patients before and after transfusion of the blood component. Scatter plots show all of the data values obtained. Data are presented as mean ± SE. *** *p* < 0.001 compared to the control group; # *p* < 0.05 compared post-versus pre-transfusion; statistically significant according to the Tukey test. pRBCs—packed red blood cells; PCs—platelet concentrates.

As shown in Figure 1, the concentration of thiols in all AML patients (before transfusion) was found to be reduced by approximately 30% (*p* < 0.001) compared to the control group. After the pRBC transfusion, a statistically significant decrease (by 18%; *p* < 0.05) in the plasma thiols was noted, compared to before transfusion (Figure 1).

**Figure 2:**
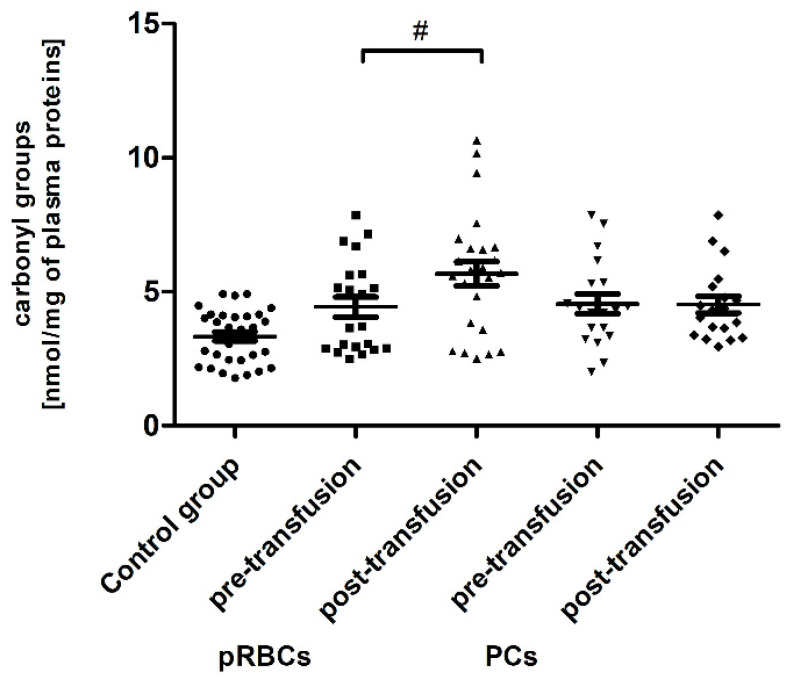
The carbonyl group content in plasma proteins in healthy volunteers (control group) and AML patients before and after transfusion of the blood component. Scatter plots show all of the data values obtained. Data are presented as mean ± SE. # *p* < 0.05 compared post- versus pre-transfusion; statistically significant according to the Tukey test. pRBCs—packed red blood cells; PCs—platelet concentrates.

**Figure 3:**
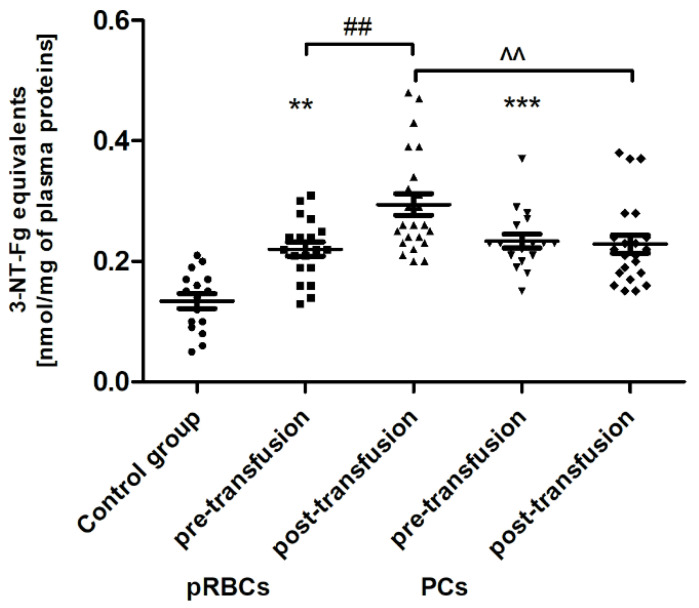
The level of 3-NT in plasma proteins in healthy volunteers (control group) and AML patients before and after transfusion of the blood component. Scatter plots show all of the data values obtained. Data are presented as mean ± SE. ** *p* < 0.01, *** *p* < 0.001 compared to the control group; ## *p* < 0.01 compared post- versus pre-transfusion; ^^ *p* < 0.01 compared post-transfusion pRBCs versus PCs; statistically significant according to the Tukey test. pRBCs—packed red blood cells; PCs—platelet concentrates.

Significant post-transfusion increases (by approximately 30 and 35%; *p* < 0.05 and *p* < 0.01, respectively) in the amount of CO groups and 3-NT level were seen only in the case of pRBC transfusion, compared to pre-transfusion values (Figure 2 and Figure 3).

Pre-transfusion (baseline) levels of 3-NT and AGE were found to be significantly higher (above 60% and 54–78%, respectively; *p* < 0.01 or *p* < 0.001), whereas the TAC of plasma and SOD activity were significantly less than in healthy volunteers. However, the baseline TAC differed significantly between the patient groups (*p* < 0.05), whereas the reduced SOD activity, compared to control, was noted only in the group receiving PLTs. Total antioxidant capacity considers the cumulative effect of all plasma antioxidants. Similarly, LDH activity (a marker of cell damage) was significantly higher (by 65%; *p* < 0.01) in AML patients prior to PC transfusion versus the volunteers. There were no significant differences in the levels of other markers between patients with AML and the control group.

**Figure 4:**
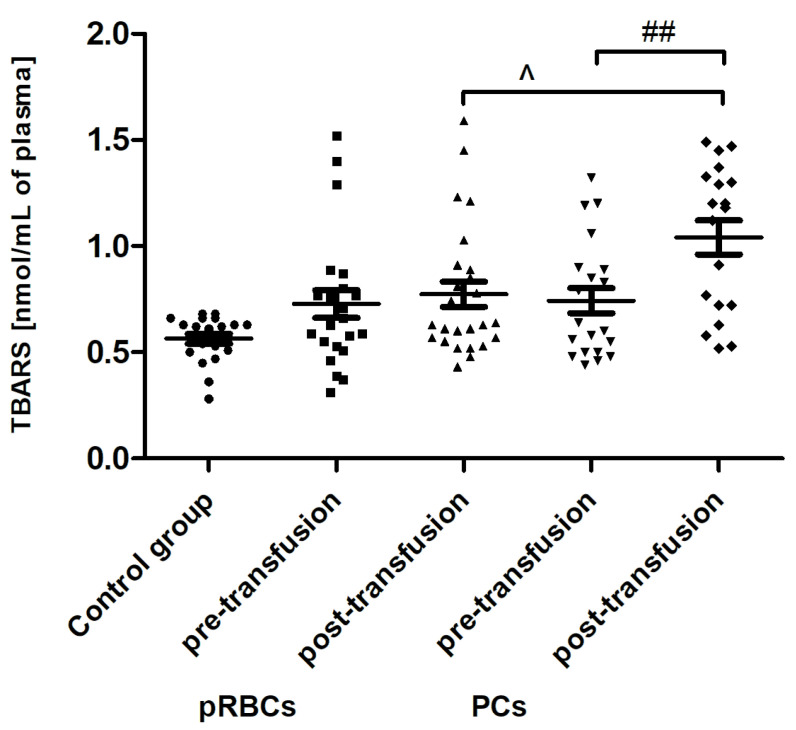
The concentration of thiobarbituric acid reactive substances (TBARS) in the plasma of healthy volunteers (control group) and AML patients before and after transfusion of the blood component. Scatter plots show all of the data values obtained. Data are presented as mean ± SE. ## *p* < 0.01 compared post- versus pre-transfusion; ^ *p* < 0.05 compared post-transfusion pRBCs versus PCs; statistically significant according to the Tukey test. pRBCs—packed red blood cells; PCs—platelet concentrates.

**Figure 5:**
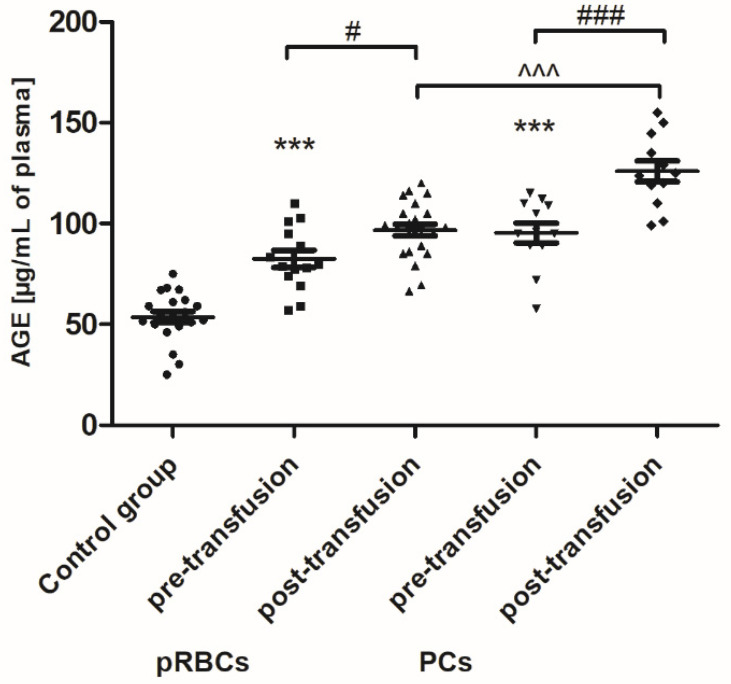
The concentration of advanced glycation end products (AGE) in the plasma of healthy volunteers (control group) and AML patients before and after transfusion of the blood component. Scatter plots show all of the data values obtained. Data are presented as mean ± SE. *** *p* < 0.001 compared to the control group; # *p* < 0.05, ### *p* < 0.001 compared post- versus pre-transfusion; ^^^ *p* < 0.001 compared post-transfusion pRBCs versus PCs; statistically significant according to the Tukey test. pRBCs—packed red blood cells; PCs—platelet concentrates.

Transfusion of the PCs, but not pRBCs, resulted in enhanced lipid peroxidation, measured by the concentration of TBARS (Figure 4). The post-transfusion increase in TBARS level (by 45%; *p* < 0.01) compared to the value before transfusion was shown. Interestingly, an increase in plasma AGE concentration, compared to that of pre-transfusion, was noted both in patients receiving pRBCs (by approximately 23%; *p* < 0.05) and those transfused with the PCs (approximately 31%; *p* < 0.001) (Figure 5).

**Figure 6:**
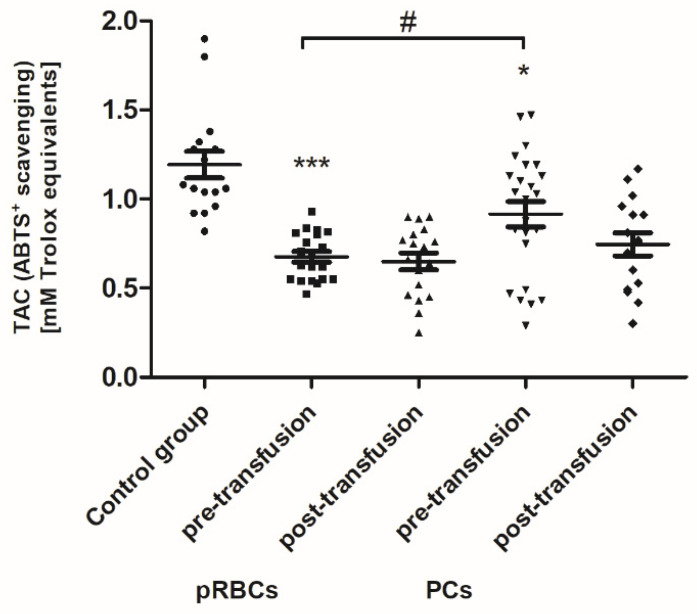
The TAC of plasma in healthy volunteers (control group) and AML patients before and after transfusion of the blood component. Scatter plots show all of the data values obtained. Data are presented as mean ± SE. * *p* < 0.05, *** *p* < 0.001 compared to the control group; # *p* < 0.05 compared pre-transfusion pRBCs versus PCs; statistically significant according to the Tukey test. pRBCs—packed red blood cells; PCs—platelet concentrates.

**Figure 7:**
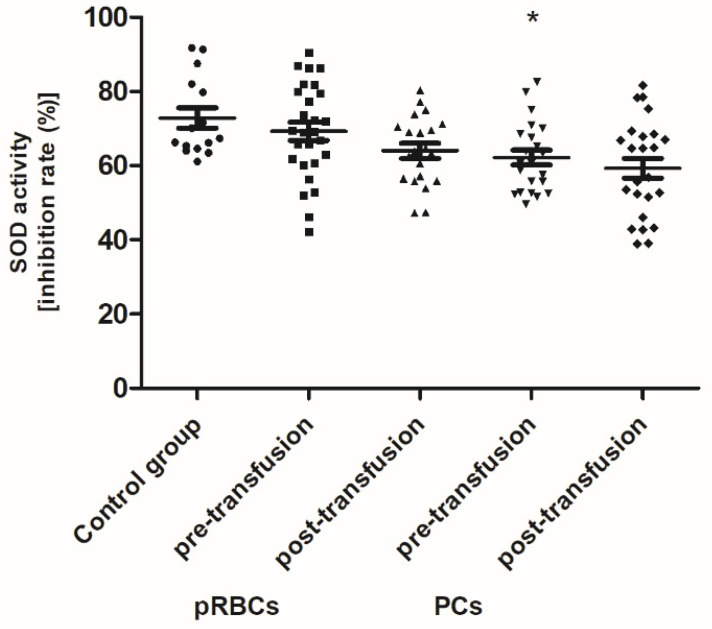
The activity of plasma SOD in healthy volunteers (control group) and AML patients before and after transfusion of the blood component. Scatter plots show all of the data values obtained. Data are presented as mean ± SE. * *p* < 0.05 compared to the control group; statistically significant according to the Tukey test. pRBCs—packed red blood cells; PCs—platelet concentrates.

**Figure 8:**
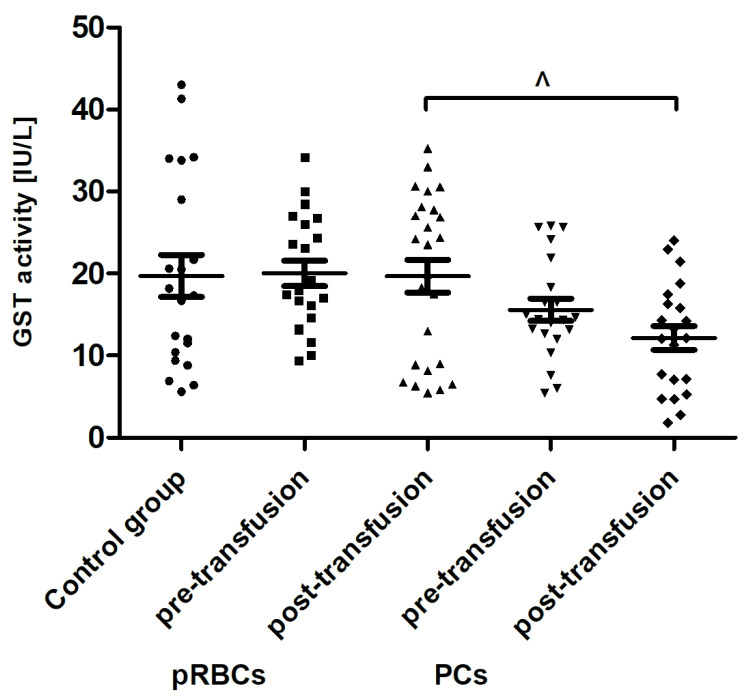
The activity of plasma GST in healthy volunteers (control group) and AML patients before and after transfusion of the blood component. Scatter plots show all of the data values obtained. Data are presented as mean ± SE. ^ *p* < 0.05 compared post-transfusion pRBCs versus PCs; statistically significant according to the Tukey test. pRBCs—packed red blood cells; PCs—platelet concentrates.

**Figure 9:**
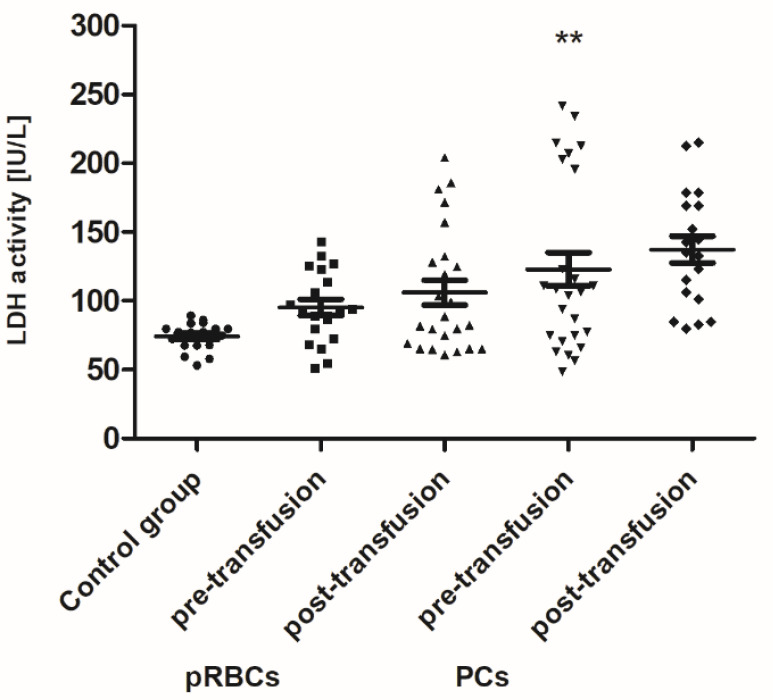
The activity of extracellular LDH in healthy volunteers (control group) and AML patients before and after transfusion of the blood component. Scatter plots show all of the data values obtained. Data are presented as mean ± SE. ** *p* < 0.01 compared to the control group; statistically significant according to the Tukey test. pRBCs—packed red blood cells; PCs—platelet concentrates.

When comparing antioxidant defense markers (TAC and activities of SOD, GST), we found that these variables showed no significant change after transfusion (neither pRBCs nor PCs) compared to these prior to transfusion (Figure 6, Figure 7 and Figure 8). Moreover, no statistically significant post- versus pre-transfusion changes in LDH activity were found (Figure 9).

## 4. Discussion

This study has evaluated the impact of pRBC transfusion on the OS level in AML patients and compared it to that of PC transfusion. We assessed a wide panel of the OS markers, such as the levels of thiols, carbonyl groups, and 3-NT (protein oxidation markers), AGE (glycation/oxidation marker), TBARS (lipid peroxidation marker), TAC, SOD, and GST (antioxidant protection markers), and LDH activity (cellular damage marker) in the blood plasma of AML patients, before and 24 h after transfusion of the blood component.

The excessive production of ROS in leukemia cells has been well documented, although the underlying mechanisms are not clearly defined [7,25]. Based on our results, an increase in OS was confirmed in AML patients compared to healthy volunteers. As a result of the neoplastic disease, protein oxidative/nitrative modifications has been shown in the patients’ plasma (Figure 1 and Figure 3). Moreover, AGE formation (Figure 5) and a significantly reduced TAC of plasma (Figure 6) has been noted in the AML group. AGE constitute a diverse group of irreversible, stable compounds, formed by binding the aldehyde group of reducing sugars with the amino group of proteins, lipids, or nucleic acids [26]. Glycation, often accompanied by lipoxidation and glycoxidation, occurs spontaneously as a non-enzymatic process. AGE by binding to a specific receptor (RAGE; receptor for advanced glycation end-products) can activate macrophages, endothelial cells, or monocytes, and consequently lead to the formation of ROS, the production of growth factors, and pro-inflammatory cytokines, and activation of transcription factors [26]. Our results clearly indicate that glycation processes are intensified in patients with AML (Figure 5). Kim et al. have shown that AGE directly induced the proliferation of primary AML cells and the human tumor cell line HEL by disrupting the signaling pathways, i.e., PI3K, MAPK, and JAK/STAT [27]. AGE have been proposed as a potential therapeutic and prognostic marker in breast cancer [28].

Recently, Tsamesidis et al. [25] also found the increased OS in the serum of AML patients, which was related to a reduction of TAC and concentration of vitamin E, and an increase in MDA and ROS levels. In our studies, no statistically significant increase in TBARS concentration (expressed in nmoles of MDA/mL of plasma) was found (Figure 4) in the AML patient group compared to healthy subjects. However, an upward trend was observed for this parameter. Moreover, trends towards decreases in the activity of antioxidant enzymes (SOD, GST) (Figure 7 and Figure 8) and LDH activity (Figure 9) in AML patients compared to volunteers were seen. SOD is the most common antioxidant enzyme, while glutathione-S-transferases belong to a super-family of phase II drug-metabolizing enzymes, important in detoxifying xenobiotics and by-products of DNA oxidation. Polymorphisms of GST genes were promising candidate biomarkers for evaluating the AML risk, which may also affect the treatment of leukemia, as GSTs have a role in detoxifying active metabolites of cytotoxic chemotherapeutic agents [29]. Lack of the significant differences between SOD, GST, and LDH in the AML compared to control groups may be a result of a large dispersion of the results due to individual differences in the studied groups, but our results remain in agreement with previous findings. Studies conducted by Rasool et al. [30] showed decreased levels of enzymatic (SOD, catalase (CAT) and glutathione peroxidase GPx)) and non-enzymatic (vitamin E and GSH) antioxidants, as well as the enhanced MDA concentration in serum of AML patients. As reported by Tsimberidou et al. [31], almost all hematological malignancies show elevated levels of serum LDH and LDH over 1.5-fold the upper limit of normal was recommended as one of the adverse independent factors predicting poorer survival in AML patients (>60 years). Generally, our results on OS level in the blood of patients with AML, compared to healthy subjects, are largely consistent with the previous data, recently reviewed in [7], and reflect the pathological state and impaired leukemia cell control.

We have found that the pRBC transfusion resulted in a significant oxidation (reflected by a reduction of –SH groups and increase of CO groups) and nitration of the AML patient plasma proteins (seen 24 h post-transfusion), while the transfusion of PC was not associated with these processes (Figure 1, Figure 2 and Figure 3). In the pool of plasma thiols measured, the free –SH group of albumin (derived from Cys-34), a dominant plasma protein (comprising above 60% of the total protein content), constitutes the vast majority; the content of low molecular weight thiols, e.g., cysteine, cysteinylglycine, glutathione, homocysteine, and γ-glutamylcysteine, is rather small in plasma [32]. The cysteinyl residue of albumin and the other thiols can be oxidized to disulfide bridges and/or other forms (i.e., sulfenate, sulfonate, or sulfonate groups). Albumin oxidation is likely related to its antioxidant properties (due to its abundance in plasma albumin is referred to as a major plasma antioxidant), where the –SH group of Cys-34 participates in free radical scavenging [33]. RBC transfusion is accompanied by transferring of many substances, i.e., “free” Hgb and its derivatives (heme, iron), proinflammatory cytokines, immunomodulatory and vasoactive mediators, proteolytic enzymes, active lipids, and microparticles, into the recipient’s circulation [3,5]. Even though the pRBCs in our study were relatively “fresh” (storage duration 2–10 days), so the storage lesion should not be abundant, it has to be pointed out that the AML patients were transfused with leukoreduced and irradiated blood components. Prestorage irradiation and filtration can cause significant damage in RBCs and intensify the RBC storage lesions, with the most obvious hemolysis increase [34]. Free Hgb and other bioactive substances comprise a source of ROS/RNS and contribute to the redox imbalance in the recipient circulation. Hgb undergoing autooxidation produces superoxide (O_2_^−•^), which in turn forms hydrogen peroxide (H_2_O_2_). Heme is an abundant source of redox-active iron that can participate in the Fenton reaction to produce toxic hydroxyl radicals [35]. The increased formation of superoxide and H_2_O_2_ also results in the oxidation of the functional ferrous Hgb to Fe(III)methemoglobin, which in turn in the two-electron oxidation by H_2_O_2_ produces oxyferrylHgb. These oxidants can not only oxidize thiols but also lead to the oxidation of the amino acid side chains in albumin and other plasma proteins. Cysteine, tyrosine, methionine, histidine, arginine, lysine, tryptophan, and proline are the most sensitive to oxidative modifications such as the introduction of CO groups (aldehyde, ketone, lactam) into the amino acid residues. In addition, OS has been shown to be associated with redox reactions that originate from Hgb reactions with nitrite and nitric oxide (NO^•^) and the resultant formation of highly toxic peroxynitrite (ONOO^−^), when NO^•^ reacts with (O_2_^−•^) released during Hgb oxidation [36]. Protein carbonylation and nitration are usually defined as irreversible post-translational modifications and can elicit conformational changes of the polypeptide chain, frequently leading to the impairment or loss of protein function, some of which contribute to altered cellular homeostasis and may underlie a number of diseases [37]. This is especially true for tyrosine nitration, since it is believed to be a vastly selective process in vitro and in vivo, preferentially directed to a subset of proteins, and within those proteins typically one or two tyrosine residues are site-specifically modified [38]. One such protein in plasma, known to be nitrated under the increased OS, is fibrinogen; the consequence of which is the alteration of fibrin structure and prothrombotic effect [39].

In line with our results, Rosa et al. [10] have shown the raise of CO groups in serum of critically ill patients after transfusion of pRBCs. In addition, in their studies protein carbonylation was positively correlated with the increased mortality. On the other hand, unlike in our study, where RBC transfusion did not cause any change in the TBARS level (Figure 4), the authors found the increased post-transfusion lipid peroxidation in critically ill patients. This discrepancy could result from significantly different experimental conditions, largely the diverse recipient groups, as well as the different measurement time (12 versus 24 h) and the type of pRBCs transfused (non-irradiated, unfiltered versus irradiated, leukocyte-reduced). In studies conducted on the preterm infants [9], the post-transfusion changes of several other oxidative stress markers, i.e., non-transferrin bound iron (NTBI), total hydroperoxides, protein thiols, and total antioxidant capacity (TAC), were found not to be clinically relevant, most likely due to too early post-transfusion sampling (after 3 h). Recently, Fernandes et al. [11] also found the elevated concentrations of TBARS, carbonyl, and –SH groups, and reduced activity of antioxidant enzymes (CAT, GPx, SOD) in recipients of multiple transfusions. Unexpectedly, the post-transfusion plasmatic SOD and GST activities of AML patients remained unchanged (Figure 7 and Figure 8), but the main forms of these enzymes are intracellularly located. The enhanced glycation processes post-transfusion of RBCs, reflected by a significantly higher AGE level (Figure 5), could be explained, at least in part, by the presence of glucose in the preservation solutions (SAGM), transfused with the blood product. It should also be emphasized that the nature of post-transfusion changes in some OS markers can be temporary. In the group of newborns receiving exchange transfusion, an increase in TBARS concentration was observed 6 h post-transfusion, but after 12 h, it reached the pre-transfusion level [40]. The 24-h post-transfusion RBC recovery is expected to average 75% in vivo, which means that the storage-damaged RBCs will be cleared from the circulation by a phagocytic system, and comprehensive studies demonstrate that most is actually cleared in the first 1–2 h [5]. Therefore, 24 h after transfusion seems optimal to assess the changes of the OS markers in the recipient’s blood.

PC transfusion, similar to pRBC transfusion, is associated with transferring into the recipient circulation of all substances accumulated during the ex vivo storage. Platelet storage lesion are mainly related to unwanted PLT activation during the preparation and storage, which result in the release of platelet-derived microparticles, an accumulation of pro-inflammatory and immunomodulatory factors, i.e., interleukine (IL)-1α, IL-6, IL-8, sCD40L, and tumor necrosis factor (TNF)-α, associated with the metabolic and morphological changes, loss of expression of the cell surface receptors, and apoptosis [4]. The ability of both resting and agonist-stimulated PLTs to the formation of ROS (i.e., superoxide anion, hydroxyl radical, hydrogen peroxide) and RFA (i.e., nitric oxide, peroxynitrite, nitroxyl), are well characterized and mainly result from the presence of mitochondria. The sources of ROS in PLTs are also NADPH oxidase (NOX), cyclooxygenase-1 (COX-1), lipoxygenase (LO), myeloperoxidase (MPO), and xanthine oxidase (XO).

Our results indicate that PC transfusion has also led to increased OS in AML patients, but it was rather due to lipid peroxidation (Figure 4), which suggests the plasma lipid damage as a consequence of this blood component transfusion. Moreover, the concentration of AGE significantly increased (24 h post-transfusion) in the plasma of AML patients, both in these receiving PCs and pRBCs (Figure 5). Significantly more AGE levels in the patient group post-PC transfusion, compared to those who received pRBC transfusions, was probably due to the fact that products might be produced by different mechanisms in either lipid peroxidation or glycoxidation reactions, respectively. Oxidative stress induces the endogenous formation and accumulation of highly reactive electrophilic aldehydes and their derivatives such as glyoxal, methylglyoxal (MG), MDA, and 4-hydroxy-2-nonenal (HNE), 4-oxo-2-nonenal (ONE), 4-hydroxy-hexanal (HHE), and acrolein, giving rise to advanced lipoxidation and glycation end products (ALE and AGE, respectively) [41]. ALE and AGE are produced from heterogeneous common precursors (i.e., glyoxal, MG) and through the same intermediates (as in the case of carboxymethyl lysine); therefore, they have the same structure, but are formed by different mechanisms. AGE represent a class of covalently modified proteins generated by oxidative and non-oxidative pathways, involving sugars and their degradation products. ALE include a variety of covalent adducts generated by the nonenzymatic reaction of reactive carbonyl species, produced by lipid peroxidation and lipid metabolism, with the nucleophilic residues of macromolecules. OS may lead to cell damage, which is manifested by the LDH release into the bloodstream. The results showed an upward trend in LDH activity in the blood plasma of AML patients compared to the control group, as well as a similar upward trend in this marker after transfusion of both blood products (Figure 9). These results imply that mechanisms such as cell damage and tissue necrosis may be responsible for the increase in plasma LDH in these patients.

## 5. Conclusions

Our results suggest that transfusion of both pRBCs and PCs result in the increased OS in AML patients; however, transfusing the former may have more severe consequences, since it is associated with the oxidative modifications of plasma proteins that are considered irreversible (such as nitration and carboxylation). Our findings shed some light on the molecular basis of the adverse post-transfusion reactions (i.e., an increased thrombosis risk after chronic RBC transfusions), and imply that the sources and mechanisms responsible for increased OS after transfusion of pRBCs and PCs may be different. These issues are worth further study. Our results should be interpreted with caution due to the relatively small size of the study groups.

## Figures and Tables

**Table 1:** Characteristics of acute myeloid leukemia (AML) patients before and 24 h after transfusion of red blood cells or platelets.

Transfused Blood Component		pRBCs *n* = 27	PCs *n* = 25
Age (years) [median (range)]		59 (26–74)	58 (37–89)
Gender (female/male)		10/17	13/12
Blood component “age” [median (range)]		6.00 (2.00–26.00)	3.00 (1.00–5.00)
PLT [×10^3^/µL]	before	30.00 (4.00–256.00)	12.00 (3.00–35.00)
(150.00–400.00) *	after	29.00 (3.00–226.00)	36.00 (2.00–88.00)
Hgb [g/dL]	before	7.50 (6.20–8.90)	8.40 (7.20–10.90)
(11.00–15.20) *	after	8.60 (6.90–10.40)	8.30 (6.5–10.5)
RBC [×10^6^/µL]	before	2.39 (1.85–2.95)	2.86 (2.24–3.49)
(3.50–5.00) *	after	2.85 (2.15–3.43)	2.82 (2.12–3.56)
WBC [×10^3^/µL]	before	0.78 (0.03–135.48)	0.49 (0.07–27.16)
(4.40–11.30) *	after	0.66 (0.03–86.27)	0.64 (0.08–19.20)
INR	before	1.23 (0.89–1.41)	1.20 (0.92–1.91)
(0.80–1.20) *	after	1.23 (0.90–1.38)	1.19 (0.88–1.54)
APTT [s]	before	31.90 (22.70–49.70)	32.90 (24.30–54.00)
(26.00–40.00) *	after	31.15 (22.80–51.30)	32.30 (24.50–53.50)
Fibrinogen [mg/dL]	before	375.00 (190.00–850.00)	351.00 (128.00–804.00)
(200.00–393.00) *	after	324.00 (176.00–894.00)	326.00 (156.00–778.00)
Ferritin [ng/mL]	before	1774.50 (688.70–7666.00)	1170.00 (199.60–1329.00)
(13.00–400.00) *	after	1720.00 (671.00–4957.00)	1185.00 (173.50–2260.00)
CRP [mg/L]	before	31.40 (1.00–386.90)	39.32 (1.97–306.90)
(0.00–5.00) *	after	32.61 (1.00–483.33)	33.80 (2.08–313.20)

The values of the determined parameters are presented as medians (range of values). APTT—the activated partial thromboplastin time; CRP—C-reactive protein; Hgb—hemoglobin; INR—normalized prothrombin time; PCs—platelet concentrates; PLT—platelets; pRBCs—packed red blood cells; RBC—red blood cells; WBC—white blood cells; *—normal ranges.

## Data Availability

The raw data analyzed during the current study are available from the corresponding author on reasonable request.
